# Связь болезни Паркинсона с метаболическими нарушениями: влияние системных дисфункций на нейродегенерацию

**DOI:** 10.14341/probl13624

**Published:** 2026-03-07

**Authors:** Ф. А. Юсупов, М. Ш. Абдыкадыров

**Affiliations:** Ошский государственный университет (ОшГУ)Кыргызстан; Osh State University (OshSU)Kyrgyzstan

**Keywords:** болезнь Паркинсона, сахарный диабет, инсулинорезистентность, диета, микробиота, митохондриальная дисфункция, окислительный стресс, Parkinson’s disease, diabetes, insulin resistance, diet, microbiota, mitochondrial dysfunction, oxidative stress

## Abstract

Данный обзор посвящен анализу взаимосвязи между болезнью Паркинсона и сахарным диабетом - двумя глобальными эпидемиями, тесно связанными со старением. Обзор опирается на новейшие эпидемиологические данные, согласно которым наличие сахарного диабета не только значительно повышает риск развития болезни Паркинсона, но и усиливает ее прогрессирование, ухудшая как моторные, так и когнитивные функции. В работе подробно рассматриваются общие патофизиологические механизмы, включающие нарушение сигнализации инсулина, развитие инсулинорезистентности, окислительный стресс, митохондриальную дисфункцию, хроническое воспаление и нейровоспаление, а также дисрегуляцию глюкозного обмена и дисбиоз кишечной микробиоты. Эти взаимосвязанные процессы формируют порочный круг, при котором каждое патологическое изменение способствует нейродегенерации, особенно в уязвимых дофаминергических нейронах черной субстанции. Особенность данного обзора заключается в интеграции современных исследований, что позволяет взглянуть на проблему двойственной природы данной коморбидности с позиции как нейродегенеративных, так и метаболических нарушений. Понимание совместных патофизиологических путей открывает перспективы для разработки многоцелевых терапевтических стратегий, включая диетические вмешательства, модификацию образа жизни и модуляцию кишечной микробиоты. Такой подход отличает данный обзор от аналогичных работ, предоставляя целостное видение проблемы и указывая на необходимость дальнейших исследований для выявления точных биомаркеров и разработки эффективных методов лечения, способных изменить течение заболевания.

## Abstract (en)

This review examines the association between Parkinson’s disease (PD) and diabetes, two global epidemics closely linked to aging. The analysis is based on recent epidemiological data indicating that diabetes significantly increases the risk of developing PD and exacerbates its progression, affecting both motor and cognitive functions. The paper explores shared pathophysiological mechanisms, including impaired insulin signaling, insulin resistance, oxidative stress, mitochondrial dysfunction, chronic inflammation and neuroinflammation, dysregulated glucose metabolism, and gut microbiota dysbiosis. These interconnected processes create a vicious cycle in which each pathology reinforces the others, driving neurodegeneration, particularly in vulnerable dopaminergic neurons of the substantia nigra. The uniqueness of this review lies in the integration of current research, providing a dual-perspective approach to comorbidity — addressing both neurodegenerative and metabolic dysfunctions. Understanding these shared mechanisms opens avenues for developing multi-targeted therapeutic strategies, including dietary interventions, lifestyle modifications, and gut microbiota modulation. This approach distinguishes the present review from similar works by offering a comprehensive perspective on the issue and emphasizing the need for further studies to identify precise biomarkers and develop effective disease-modifying treatments.

## Введение

Болезнь Паркинсона (БП) представляет собой прогрессирующее нейродегенеративное заболевание, характеризующееся дегенерацией дофаминергических нейронов в черной субстанции и накоплением внутриклеточных включений — телец Леви [1][2]. Развитие патологического процесса обусловлено комплексными молекулярными нарушениями, включая агрегацию токсичных форм α-синуклеина (α-Syn), дисфункцию митохондрий, энергетическую недостаточность, хроническое нейровоспаление, окислительный стресс и влияние экзогенных факторов, таких как воздействие пестицидов и других нейротоксинов [3]. Генетическая предрасположенность также играет значительную роль, модулируя уязвимость к этим патогенетическим механизмам. У разных пациентов ведущие механизмы могут варьироваться, а их сочетание эволюционирует с прогрессированием заболевания. Накапливающиеся данные указывают на тесную взаимосвязь между патогенезом БП и системными метаболическими нарушениями, включая инсулинорезистентность и сахарный диабет.

Сахарный диабет (СД) представляет собой гетерогенную группу метаболических заболеваний, характеризующихся устойчивой гипергликемией, обусловленной нарушением секреции инсулина, снижением чувствительности периферических тканей к его воздействию или сочетанием этих факторов [4]. Согласно прогнозам ВОЗ, к 2051 г. глобальное количество пациентов с данным заболеванием может достигнуть 1,3 млрд человек [5]. В Российской Федерации за последние три десятилетия суммарное бремя болезни, связанное с СД, увеличилось на 206% [6]. Одновременно наблюдается рост показателей смертности от его осложнений, которые составляют 671 случай на 100 тыс. населения [7]. Длительное нарушение гомеостаза глюкозы приводит к структурным и функциональным повреждениям различных органов и систем, включая сетчатку глаза, почки, сердечно-сосудистую систему, а также периферическую и центральную нервную систему, что в итоге способствует прогрессированию системных патологических процессов [8].

Сахарный диабет 2 типа (СД2) является наиболее распространенной формой заболевания и развивается вследствие сочетанной патологии углеводного обмена, включающей инсулинорезистентность и относительную недостаточность инсулиновой секреции. В ряде случаев ведущим механизмом патогенеза выступает первичное нарушение функции β-клеток поджелудочной железы с последующей инсулинорезистентностью или без нее, что приводит к прогрессирующему дисбалансу метаболической регуляции и развитию системных осложнений [4].

Метаболическое и нейродегенеративное нарушения представляют собой два тесно взаимосвязанных аспекта старения, с особым акцентом на СД и болезнь Паркинсона как наиболее распространенные и социально значимые состояния. В условиях глобального увеличения продолжительности жизни отмечается неуклонный рост заболеваемости этими патологиями, что приводит к серьезным вызовам для систем здравоохранения. В последние годы усилилось внимание к молекулярным механизмам, связывающим нарушение инсулиновой сигнализации, метаболические дисфункции и дегенеративные процессы в головном мозге. Наблюдаемое перекрестное влияние диабета и нейродегенерации может усугублять клиническое течение каждого заболевания, осложняя диагностику и терапию. При этом учитываются не только параллельные патофизиологические процессы, но и потенциальные синергетические механизмы, способные усиливать воспалительные и дегенеративные изменения. Таким образом, в контексте стареющего населения все более актуальной становится необходимость интегрированного подхода к профилактике и лечению — с акцентом на раннее вмешательство, до наступления необратимых нейрональных потерь.

## Цель данного обзора

Систематизировать современные данные (2020–2025 гг.) о коморбидности болезни Паркинсона и сахарного диабета, выявить общие патофизиологические механизмы и оценить их клинические и терапевтические последствия. Особое внимание уделяется метаболическим нарушениям, таким как инсулинорезистентность, окислительный стресс, митохондриальная дисфункция и нейровоспаление.

## Материалы и методы

Настоящая работа представляет собой детальный обзор научных публикаций, посвященных взаимосвязи между болезнью Паркинсона и сахарным диабетом. Анализ литературы проводился на основе исследований, опубликованных в ведущих научных журналах преимущественно в период 2020–2025 гг. Поиск источников осуществлялся в специализированных базах данных, включая PubMed, Embase, Scopus, Cochrane и Google Scholar. Основное внимание уделено эпидемиологическим аспектам, общим патофизиологическим механизмам и возможным терапевтическим стратегиям.

## Критерии включения и выключения

Включенные в обзор исследования представлены рецензируемыми научными публикациями, вышедшими в основном в период 2020–2025 гг., посвященным эпидемиологическим аспектам коморбидности болезни Паркинсона и сахарного диабета. Рассматриваются работы, изучающие ключевые патофизиологические механизмы этой взаимосвязи, включая инсулинорезистентность, окислительный стресс, митохондриальную дисфункцию, нейровоспаление, аномальную агрегацию белков, дисрегуляцию метаболизма глюкозы и нарушение состава кишечной микробиоты.

Обзор исключает работы, опубликованные вне установленного временного диапазона, за исключением тех, которые являются фундаментальными для рассматриваемого вопроса. Не включаются нерецензируемые источники, такие как материалы конференций, препринты без экспертной оценки или публикации, не соответствующие академическим стандартам. Кроме того, исключены исследования, не имеющие непосредственного отношения к коморбидности болезни Паркинсона и сахарного диабета, либо рассматривающие одно из заболеваний без учета их патогенетической взаимосвязи.

## Эпидемиологическая связь между диабетом и болезнью Паркинсона

Накопленные эпидемиологические данные, в основном полученные из когортных исследований и метаанализов, убедительно указывают на то, что СД2 значительно увеличивает риск развития спорадической БП (табл. 1) [1]. Метаанализ семи когортных исследований показал, что у пациентов с диабетом риск развития БП на 38% выше по сравнению с недиабетиками, при этом у женщин риск выше на 50%, а у мужчин — на 40% [9]. Крупномасштабное когортное исследование, проведенное в 2020 г. с участием более 15 миллионов человек, выявило значительное повышение риска развития БП по мере увеличения длительности СД и выраженности гипергликемии [1]. В исследовании использовался показатель отношения рисков (ОР), демонстрирующий, насколько вероятность возникновения заболевания (в данном случае БП) выше среди пациентов с диабетом по сравнению с недиабетической популяцией. Значение ОР выше 1 свидетельствует о повышенном риске, тогда как ОР ниже 1 — о его снижении. Согласно полученным данным, ОР для БП составлял 1,185 при длительности СД менее 5 лет и увеличивался до 1,618 при диабете продолжительностью более 5 лет, подтверждая кумулятивное негативное воздействие хронических метаболических нарушений [1]. Эти результаты подчеркивают, что риск БП возрастает не просто из-за наличия диабета, а в зависимости от его длительности и тяжести метаболической дисрегуляции. Данный эффект носит дозозависимый и временно-зависимый характер, при котором стойкое нарушение метаболического баланса способствует прогрессирующему повреждению нервной системы, повышая ее восприимчивость к нейродегенерации. Следовательно, своевременная диагностика и агрессивное лечение диабета, включая управление предиабетическими состояниями, являются ключевыми стратегиями для потенциального смягчения или замедления развития БП, что подчеркивает необходимость ранних терапевтических вмешательств.

**Table:** Таблица 1. Сводка последних эпидемиологических исследований (2020–2025 гг.) по связи СД/СД2 и риску/прогрессированию БП

Исследование (год)	Тип исследования	Размер популяции. Случаи	Ключевые выводы (отношение рисков/шансов с ОР)	Особенности
2020 [1]	Когортное	>15 млн субъектов, 31,577 случая БП	ОР (СД<5 лет) = 1,185 (1,143–1,229); ОР (СД≥5 лет) = 1,618 (1,566–1,672); ОР (НГН) = 1,038 (1,009–1,067)	Риск БП значительно увеличивается с продолжительностью СД и гипергликемией
2020 [11]	Когортное	2,362,072 индивида с СД2	ОР (1 параметр тяжести СД) = 1,09 (1,04–1,15); ОР (6 параметров тяжести СД) = 2,78 (2,05–3,79)	Тяжесть СД положительно ассоциируется с риском развития БП
2021 [10]	Метаанализ	Не указано	ОР (любой СД) слегка увеличивает риск БП; ОР (СД2) = 1,21 (1,07–1,36)	СД2 связан с повышенным риском БП и более быстрым прогрессированием моторных и когнитивных симптомов
2022 [9]	Метаанализ	29,9 млн участников, 86,345 случая БП	ОР (СД) = 1,27 (1,20–1,35); ОР (предиабет) = 1,04 (1,02–1,07)	Пациенты с СД имеют на 27% повышенный риск БП; с предиабетом — на 4%
2023 [14]	Проспективное	23,901 индивида, 213 случаев БП	ОР (СД2) = 1,85 (1,24–2,76)	СД2 является фактором риска развития БП
2024 [15]	Когортное	89,074 бенефициара	ОР (GLP-1RA vs DPP4i) = 0,77 (0,63–0,95)	Использование агонистов GLP-1R связано со снижением риска БП у пациентов с СД2

## Роль предиабета в повышении риска болезни Паркинсона

Предиабет, характеризующийся нарушением гликемии натощак (НГН), также связан с умеренно повышенным риском БП (ОР=1,038 для НГН) [1]. Недавний метаанализ 15 когортных исследований (29,9 миллиона участников) выявил увеличение риска БП на 4% у пациентов с предиабетом по сравнению с лицами с нормальным уровнем глюкозы в крови [9]. Тот факт, что предиабет и даже нарушение гликемии натощак уже увеличивают риск БП, в сочетании с тем, что у пациентов с БП часто наблюдаются аномалии метаболизма глюкозы еще до постановки официального диагноза диабета [9], указывает на длительную продромальную фазу, в течение которой метаболическая дисрегуляция незаметно способствует нейродегенерации. Это говорит о важности регулярного рутинного метаболического скрининга у лиц с риском БП, а также у пациентов с БП, поскольку забота о метаболическом здоровье может сыграть ключевую роль еще до появления явных симптомов болезни и серьезного повреждения нервных клеток.

Помимо начала заболевания, СД2 также может влиять на прогноз и прогрессирование симптомов БП [1]. СД2 ассоциируется с более быстрым прогрессированием как моторных симптомов (о чем свидетельствуют более высокие баллы по шкале UPDRS), так и когнитивного снижения (измеряемого снижением баллов по Монреальской шкале когнитивной оценки) [10]. Тяжесть диабета, оцениваемая по таким параметрам, как количество пероральных гипогликемических препаратов, продолжительность диабета, использование инсулина или наличие хронических осложнений (например, хроническая болезнь почек, диабетическая ретинопатия), положительно коррелирует с повышенным риском развития БП [11]. Пациенты с большим количеством параметров тяжести диабета демонстрировали прогрессивно более высокое ОР для БП (например, ОР 1,55 для трех параметров, ОР 1,96 для четырех параметров, ОР 2,78 для шести параметров) [11]. Гликемическая вариабельность, а не только постоянно высокий уровень глюкозы, может быть особенно пагубной для диабетических пациентов, усугубляя окислительный стресс, воспаление и повреждение эндотелия, что потенциально увеличивает восприимчивость к БП [12].

## Методологические особенности и противоречивые результаты исследований

Хотя большинство проспективных когортных исследований подтверждают, что СД является фактором риска БП, некоторые ретроспективные исследования случай-контроль показали противоречивые результаты, иногда указывая на снижение заболеваемости БП у пациентов с СД [10]. Это расхождение может быть связано с методологической гетерогенностью, смешивающими факторами и систематическими ошибками, такими как ошибки включения и отзыва, присущие различным методам исследований [10]. Например, диагностика СД2, основанная исключительно на самоотчете в некоторых исследованиях, может привести к недооценке или неправильной классификации [13].

Сильная эпидемиологическая связь между БП и СД указывает на общие базовые патологические пути. Эти общие механизмы включают сложное взаимодействие клеточных и молекулярных дисфункций, которые в совокупности способствуют нейродегенерации.

## Дисфункция инсулиновой сигнализации и инсулинорезистентность

Передача сигналов инсулина в мозге имеет решающее значение для выживания нейронов, их пластичности, регуляции окислительного стресса и контроля нейровоспаления [1]. Инсулинорезистентность (ИР), состояние, при котором клетки не реагируют нормально на инсулин, является характерным признаком СД2 и ключевой патологической особенностью, все чаще признаваемой при нейродегенеративных расстройствах, таких как БП и болезнь Альцгеймера (БА) [10][16]. ИР проявляется нарушением поглощения глюкозы, повышенным уровнем сахара в крови и нарушением липидного обмена, часто сопровождаясь стойким низкоинтенсивным системным воспалением [17]. Нейропатологические исследования показали, что инсулиновые рецепторы плотно экспрессируются на дофаминергических нейронах компактной части черной субстанции (SNpc), основного места нейродегенерации при БП. У пациентов с БП наблюдалась потеря иммунореактивности и мРНК инсулиновых рецепторов в SNpc [10]. Дисфункция ключевых путей передачи сигналов инсулина, может приводить к усилению фосфорилирования тау-белка и ускорению агрегации α-Syn, что является критически важными событиями в патогенезе БП. ИР мозга предлагается в качестве основного фактора, способствующего более быстрому прогрессированию заболевания, наблюдаемому у пациентов с БП и сопутствующим СД2. ИР, вызванная диетой с высоким содержанием жиров у животных, усиливает потерю дофамина и ухудшает двигательные функции после воздействия нейротоксичных веществ [10].

## Окислительный стресс

Окислительный стресс, дисбаланс между образованием активных форм кислорода (АФК) и антиоксидантной защитой, является общим патологическим путем, связывающим БП и СД2 [10]. Дофаминергические нейроны в черной субстанции особенно уязвимы к окислительному стрессу из-за их внутренних характеристик, включая высокое содержание железа, высокую метаболическую активность и ограниченную антиоксидантную защиту [18]. Гипергликемия, центральная особенность СД, напрямую способствует окислительному стрессу. Увеличение внутринейронального потока глюкозы приводит к перегрузке митохондриальной электронно-транспортной цепи (ЭТЦ), что приводит к чрезмерному образованию АФК [18]. АФК наносят ущерб жизненно важным макромолекулам, таким как белки, липиды и ДНК. Повреждение ДНК, вызванное окислительным стрессом, в частности разрывы цепей, запускает активацию поли (АДФ-рибоза) полимеразы 1 (PARP1), никотинамидадениндинуклеотид (НАД+) -зависимого фермента репарации ДНК. Активация PARP1, совместно с полиоловым путем, истощает внутриклеточный НАД+. Это ингибирование гликолиза приводит к накоплению предшественников конечных продуктов гликирования (КПГ), создавая самоподдерживающийся цикл окислительного повреждения [18]. Полиоловый путь, альтернативный путь метаболизма глюкозы, активируемый при гипергликемии, еще больше усугубляет окислительный стресс, потребляя НАДФН (необходимый для антиоксидантной защиты) и НАД+ [18].

## Митохондриальная дисфункция

Митохондриальная дисфункция является фундаментальным и общим механизмом в патогенезе и прогрессировании как БП, так и СД2 [2]. При БП многие семейные гены, связанные с БП (например, SNCA, LRRK2, Parkin, PINK1, DJ-1), непосредственно участвуют в поддержании функции митохондрий и контроле качества [10]. Например, α-Syn может взаимодействовать с митохондриальной мембраной, вызывая токсичность, в то время как мутации в PINK1 и Parkin нарушают митофагию, приводя к накоплению поврежденных митохондрий [10]. При СД2 дисфункциональные митохондрии в панкреатических β-клетках нарушают выработку и секрецию инсулина [19]. Исследования показывают, что повреждение митохондрий может запускать стрессовую реакцию, которая влияет на созревание и функцию β-клеток [19]. Митохондрии являются основными местами производства АФК и имеют решающее значение для клеточной энергии, метаболизма и окислительно-восстановительного гомеостаза. Митохондриальная дисфункция напрямую способствует инсулинорезистентности, активируя фосфорилирование белка IRS, что еще больше нарушает передачу сигналов инсулина [10]. Гипергликемия напрямую повреждает митохондрии, приводя к нарушению окислительного фосфорилирования и снижению синтеза АТФ, что особенно пагубно для энергоемких дофаминергических нейронов [18].

## Хроническое низкоинтенсивное воспаление

Хроническое низкоинтенсивное воспаление, часто называемое «сеновоспалением» при старении, является значительным фактором, способствующим как СД, так и БП [1]. Метаболические расстройства, включая инсулинорезистентность, часто связаны с провоспалительным состоянием, характеризующимся повышенными уровнями воспалительных цитокинов, таких как интерлейкин-6 (ИЛ-6) и фактор некроза опухоли-альфа (ФНО-α). В центральной нервной системе (ЦНС) нейровоспаление, опосредованное активными клетками микроглии, играет ключевую роль в дегенерации дофаминергических нейронов при БП [10]. Метаболические нарушения могут смещать микроглии от нейропротекторного (M2) к нейротоксическому (M1) фенотипу [10]. Путь инфламмасомы NLRP3 является ключевым медиатором хронического системного и мозгового воспаления, связывая СД и БП. Его активация приводит к выработке провоспалительных цитокинов, таких как ИЛ-1β и ИЛ-18, которые нарушают функцию панкреатических β-клеток, вызывают апоптоз клеток и усугубляют инсулинорезистентность. Повреждение митохондрий, включая накопление поврежденных митохондрий и увеличение митохондриальных АФК из-за ингибирования митофагии, играет критическую роль в активации инфламмасомы NLRP3 [10].

## Накопление патологических белков

Накопление неправильно свернутых белков является общим патологическим путем, характерным как для БП, так и для СД2 [10]. При БП агрегация α-Syn в тельца и нейриты Леви является патологическим признаком, приводящим к прогрессирующей гибели нейронов [1]. При СД2 полипептид островковых амилоидов (IAPP), также известный как амилин, образует фибриллярные агрегаты в панкреатических островках, способствуя дисфункции и гибели β-клеток [10]. Важно отметить, что существует описанное перекрестное взаимодействие между ними. Эксперименты in vitro показывают, что амилоиды IAPP способствуют агрегации α-Syn, и их совместная агрегация происходит быстрее, чем агрегация каждого белка по отдельности [10]. Увеличение агрегации и фосфорилирования α-Syn с частичной ко-локализацией с IAPP наблюдалось в поджелудочной железе и мозге животных моделей СД2, что указывает на продромальные изменения, связанные с БП [10]. Гипергликемия и хроническая дисрегуляция глюкозы способствуют гликированию белков, включая α-Syn, продуктами, такими как метилглиоксаль (MGO). Гликирование α-Syn ускоряет его пагубные эффекты и способствует его накоплению, образуя молекулярную связь между СД2 и БП. У пациентов с диабетом без клинических признаков болезни Паркинсона было обнаружено снижение связывания DAT в стриатуме и повышение уровня α-Syn и тау-белка в спинномозговой жидкости, что дополнительно связывает метаболическую дисфункцию с патологией БП [1].

Нарушения системного и регионального метаболизма глюкозы наблюдаются у пациентов с БП на всех стадиях заболевания, влияя на заболеваемость, прогрессирование и специфические фенотипы [1]. Глюкоза является основным энергетическим субстратом для мозга взрослого человека, и ее активное окисление производит АТФ, жизненно важный для функции нейронов [9]. Дисфункция метаболизма глюкозы нарушает нормальное функционирование нейронов, что широко наблюдается при нейродегенеративных заболеваниях [1]. Механизмы, способствующие дисрегуляции глюкозы при БП, включают инсулинорезистентность, окислительный стресс, аномальную гликированную модификацию, дисфункцию гематоэнцефалического барьера и повреждения, вызванные гипергликемией [20]. Эти нарушения могут приводить к чрезмерному производству MGO и АФК, нейровоспалению, аномальной агрегации белков, митохондриальной дисфункции и снижению уровня дофамина, что в конечном итоге приводит к недостаточности энергоснабжения и дисрегуляции нейромедиаторов [20]. Примечательно, что у пациентов с БП сверхэкспрессия гексокиназы 2 (HK2) в области черной субстанции среднего мозга была связана с усилением гликолиза, что приводило к увеличению выработки лактата и последующей гибели дофаминергических нейронов [20].

## Дисбиоз кишечной микробиоты и ось «Кишечник-Мозг»

Дисбиоз кишечной микробиоты и ось «кишечник-мозг». Появляющиеся данные подчеркивают критическую роль дисбиоза кишечной микробиоты как при метаболических расстройствах, так и при нейродегенеративных заболеваниях [10]. Дисбиоз при СД2 характеризуется дефицитом полезных короткоцепочечных жирных кислот (КЦЖК) и измененным соотношением Firmicutes к Bacteroidetes, часто ассоциирующимся с хроническим воспалением [10]. Аналогично у пациентов с БП наблюдается измененный состав кишечной микробиоты с увеличением численности некоторых бактерий (например, Bacteroidetes, Verrucomicrobia) и снижением уровня бактерий, продуцирующих КЦЖК (например, Blautias spp., Coprococcus spp., Roseburia spp.) [10]. Ось «кишечник-мозг» обеспечивает важную связь: вредные метаболиты и провоспалительные цитокины, образующиеся при дисбиозе кишечника, могут нарушать целостность кишечного барьера, приводя к системному воспалению и дисфункции панкреатических β-клеток. Эти воспалительные сигналы затем могут пересекать гематоэнцефалический барьер, способствуя нейровоспалению, активации иммунитета и окислительному стрессу в мозге [10]. Липополисахариды (ЛПС), продуцируемые бактериями, которые взаимодействуют с толл-подобными рецепторами (TLR), опосредуя воспалительную реакцию, обнаруживаются в повышенных уровнях в крови пациентов с СД и нейродегенеративными заболеваниями, такими как БП [10]. Примечательно, что желудочно-кишечные осложнения и дисбиоз, связанный с микробиотой, могут предшествовать моторным симптомам БП, что предполагает, что кишечник является потенциальным ранним местом патологии [10]. Эти механизмы не действуют изолированно; они образуют сложный, взаимосвязанный порочный круг, где, например, ИР ведет к окислительному стрессу, митохондриальной дисфункции и хроническому воспалению [10]. В свою очередь эти состояния усугубляют неправильное сворачивание белков и далее нарушают передачу сигналов инсулина. Это означает, что повреждение одной части цикла может быть недостаточным, если другие усиливающие петли остаются активными, что подчеркивает необходимость системного подхода к вмешательству. Хотя метаболические нарушения носят системный характер, дофаминергические нейроны в черной субстанции непропорционально страдают [18]. Эта избирательная уязвимость объясняется их уникальными физиологическими характеристиками, включая высокое содержание железа, высокую потребность в энергии, низкий уровень антиоксидантной защиты и автономную пейсмейкерную активность [18]. Это означает, что системное метаболическое воздействие (например, гипергликемия или ИР) действует как «второй удар» по уже изначально восприимчивой популяции нейронов, выталкивая их за пределы критического порога выживания. Это объясняет, почему не у всех пациентов с диабетом развивается БП, и почему мозг, особенно черная субстанция, является мишенью. Кроме того, дисбиоз кишечной микробиоты, присутствующий как при СД, так и при БП, позволяет воспалительным медиаторам и метаболитам, происходящим из кишечника, напрямую влиять как на функцию панкреатических β-клеток, так и на здоровье мозга. Это указывает на то, что кишечник является не просто периферическим симптомом, а центральным игроком в коморбидности, действуя как «конвергентный центр», где метаболические и нейродегенеративные патологии инициируются или усугубляются. Это открывает новые, многообещающие пути для терапевтических вмешательств, направленных на микробиом кишечника (например, пробиотики, диетические изменения, трансплантация фекальной микробиоты) не только для желудочно-кишечных симптомов, но и в качестве стратегии модификации заболевания как при диабете, так и при БП.

## Генетическая предрасположенность и общая восприимчивость

Как БП, так и диабет могут иметь генетический компонент, причем семейные случаи БП связаны со специфическими мутациями генов (например, SNCA, GBA, LRRK2, PARK2, PINK1, VPS35) [10]. Хотя прямые общие генетические факторы риска, явно идентифицированные для обоих состояний в представленных данных, ограничены, общие молекулярные пути (например, митохондриальная дисфункция, передача сигналов инсулина) убедительно указывают на потенциальные генетические совпадения. Например, несколько генов, связанных с БП (PINK1, Parkin, LRRK2), как известно, участвуют в функции митохондрий и окислительном фосфорилировании, которые также являются центральными для метаболического здоровья и патогенеза диабета. Концепция «взаимодействия ген-среда» имеет решающее значение для спорадической БП, где уникальные генотипы взаимодействуют с факторами окружающей среды, влияя на фенотип заболевания [21]. Этот принцип, вероятно, распространяется и на коморбидность с диабетом, когда генетическая предрасположенность к метаболической дисфункции может сделать индивидов более восприимчивыми к экологическим триггерам, которые также влияют на нейродегенерацию. Это указывает на возможность персонализированных подходов в медицине, где генетическое скринингование на предрасположенность к метаболическим нарушениям или путям, связанным с БП, может помочь в разработке ранних, целенаправленных вмешательств в образ жизни для предотвращения или задержки начала заболевания у лиц из группы риска.

## Экологические, диетические факторы и факторы образа жизни

## Диетические факторы

Нездоровое питание, особенно характерное для «западной диеты» (высокое потребление красного мяса, обработанных продуктов, насыщенных жиров и рафинированных углеводов), связано с повышенным риском нейродегенеративных и метаболических заболеваний, включая БП и СД. В частности, избыточное потребление добавленных сахаров ассоциируется с повышенной распространенностью БП, особенно среди женщин, злоупотребляющих алкоголем, и лиц с диабетом [22]. Это свидетельствует о том, что добавленный сахар выступает не только независимым фактором риска, но и усиливает патологическую взаимосвязь между диабетом и БП, способствуя прогрессированию нейродегенеративных процессов [22]. В то же время диетические модификации признаются эффективной стратегией профилактики и лечения данных заболеваний. Средиземноморская диета, богатая антиоксидантами, ненасыщенными жирами и клетчаткой, обладает выраженным нейропротекторным действием и может замедлять развитие и прогрессирование БП. Сбалансированное питание, стимулирующее выработку КЦЖК и поддерживающее разнообразие кишечной микробиоты, также способствует защите нервной системы. В качестве потенциальных терапевтических стратегий рассматриваются пробиотики, синбиотики и трансплантация фекальной микробиоты (ТФМ), направленные на восстановление микробиомного баланса и снижение риска БП [23]. Это подчеркивает важность комплексного подхода, включающего коррекцию питания и изменение образа жизни, для предотвращения или смягчения комбинированного риска нейродегенерации и метаболических нарушений [24].

Недостаток физической активности является общим фактором риска как для сахарного диабета, так и для болезни Паркинсона [25].

## Экологические токсины

Пестициды и тяжелые металлы являются известными экологическими факторами риска БП, способствующими митохондриальной дисфункции, окислительному стрессу и нарушению деградации белков [26]. Хотя в представленных данных они явно не связаны с диабетом, их широкое клеточное воздействие предполагает потенциальные синергетические эффекты с метаболическими стрессорами. Эктопическое отложение жира, особенно в печени, усиливает системное воспаление и метаболический стресс, усиливая воспалительные процессы и способствуя накоплению поврежденных или неправильно свернутых белков, связывая метаболический синдром с такими состояниями, как БП [27].

## Социально-экономические факторы и урбанизация

Индустриализация, урбанизация и социально-экономический прогресс связаны с увеличением распространенности БП [25], потенциально из-за увеличения воздействия экологических токсинов и изменений образа жизни, связанных с современным обществом.

Признание того, что множество взаимосвязанных путей способствуют коморбидности, логически приводит к выводу, что монотерапия может быть недостаточной. Акцент на образе жизни и здоровье кишечника наряду с фармакологическими агентами указывает на переход к более целостным и многоцелевым подходам. Будущие исследования и клиническая практика, вероятно, будут сосредоточены на мультицелевые вмешательства, которые одновременно воздействуют на несколько патологических механизмов, потенциально интегрируя фармакологическое лечение с персонализированными диетическими вмешательствами и изменениями образа жизни. Это также подчеркивает важность раннего вмешательства, возможно, даже в досимптомной фазе, до того как произойдет необратимая нейродегенерация.

## Заключение

Связь между БП и СД, особенно СД2, является растущей проблемой общественного здравоохранения, обусловленной общими эпидемиологическими тенденциями и сложными, взаимосвязанными патофизиологическими механизмами. Эпидемиологические данные убедительно демонстрируют, что СД2 значительно увеличивает риск развития БП и усугубляет ее прогрессирование, причем этот риск возрастает с увеличением продолжительности и тяжести диабета. Даже предиабет связан с повышенным риском БП, что указывает на длительную продромальную фазу, в течение которой метаболическая дисрегуляция незаметно способствует нейродегенерации. В основе этой коморбидности лежит сложная сеть общих молекулярных и клеточных дисфункций. Нарушение передачи сигналов инсулина и ИР, окислительный стресс, митохондриальная дисфункция, хроническое воспаление и нейровоспаление, а также накопление неправильно свернутых белков образуют порочный круг, где каждая патология усиливает другие. Дофаминергические нейроны черной субстанции, изначально уязвимые, особенно восприимчивы к этим системным метаболическим воздействиям. Ось «кишечник-мозг» также выступает в качестве центрального патогенного узла, где дисбиоз кишечной микробиоты способствует как метаболическим, так и нейродегенеративным процессам. Это подчеркивает сложность трансляции доклинических результатов в клиническую практику и указывает на необходимость более глубокого понимания патофизиологии БП и разработки более целенаправленных и/или многокомпонентных терапий. Будущие направления исследований должны сосредоточиться на разработке многоцелевых терапевтических стратегий, которые одновременно воздействуют на несколько патологических путей, включая фармакологические агенты, улучшающие метаболическую функцию, и вмешательства в образ жизни, такие как диетические модификации (например, средиземноморская диета) и модуляцию кишечной микробиоты (например, пробиотики, ТФМ). Выявление точных биомаркеров для ранней диагностики и мониторинга прогрессирования заболевания, а также лучшее понимание двунаправленной природы связи между СД и БП, будут иметь решающее значение для разработки эффективных методов лечения, изменяющих течение заболевания, и улучшения результатов лечения пациентов.

## ДОПОЛНИТЕЛЬНАЯ ИНФОРМАЦИЯ

Источники финансирования. Работа выполнена по инициативе авторов без привлечения финансирования.

Конфликт интересов. Авторы декларируют отсутствие явных и потенциальных конфликтов интересов, связанных с содержанием настоящей статьи.

Участие авторов. Все авторы одобрили финальную версию статьи перед публикацией, выразили согласие нести ответственность за все аспекты работы, подразумевающую надлежащее изучение и решение вопросов, связанных с точностью или добросовестностью любой части работы.
